# Anomalous gallbladder septum—A case report

**DOI:** 10.1016/j.ijscr.2021.106082

**Published:** 2021-06-10

**Authors:** P. Bamback, K.C. Baumgardner, M. Bartanuszova, H.L. Nation, A.P. Occhialini

**Affiliations:** a Long School of Medicine, University of Texas-Health at San Antonio, 7703 Floyd Curl Dr., San Antonio, TX 78229, United States of America; b Department of Cell Systems and Anatomy, University of Texas-Health at San Antonio, 7703 Floyd Curl Dr., San Antonio, TX 78229, United States of America

**Keywords:** Case report, Gallbladder, Gallbladder septum, Gallbladder valve, Anomalous hepatic artery, mm, millimeters, ml, milliliters, TS, transverse septum, GB, gallbladder, CD, cystic duct, HD, hepatic duct, CBD, common bile duct, RHA, right hepatic artery, LHA, left hepatic artery, ALHA, accessory left hepatic artery, CHA, common hepatic artery, CA, cystic artery, SMA, superior mesenteric artery, CT, celiac trunk, SA, splenic artery, LGA, left gastric artery, GDA, gastroduodenal artery, D, duodenum

## Abstract

**Introduction:**

Anomalies and diseases of the biliary system are common with over 20 million cases of biliary disease and an estimated 1.8 million ambulatory visits each year in the United States. Congenital anomalies of the gallbladder are rare and include complete and partial duplications, floating gallbladders, and agenesis. Septations have also been reported in the literature. Case reports have typically described these as longitudinal. Transverse septa, when reported, are associated with inflammation or cholelithiasis. Variations in the cystic duct and vasculature in the portal triad have also been well described.

**Presentation of case:**

During the dissection of a 91-year-old female cadaver, an enlarged gallbladder with a partial transverse septum was observed. The gallbladder contained approximately 350 ml of bile, no stones, and had a partial transverse septum near the infundibulum. The hepatic, cystic, and common bile ducts were enlarged, but of normal configuration. Vascular anomalies were also present, including an accessory left hepatic artery from the left gastric artery and an anomalous origin of the right hepatic artery from the superior mesenteric artery.

**Discussion:**

This is the first described case of a partial transverse septum with a markedly enlarged gallbladder, dilated duct system, and vascular anomalies in a patient with no evidence of gallstones, inflammation, or scarring.

**Conclusion:**

With the prevalence of biliary disease and frequent subsequent surgery it is essential to appreciate all anatomical variations to avoid iatrogenic injuries to these structures during surgery.

## Highlights

• Report of a partial transverse gallbladder septum.
• Report includes a dilated biliary system.
• Report of two vascular anomalies.

## 1 Introduction

With over 20 million cases of biliary disease and an estimated 1.8 million ambulatory visits each year in the United States, it is essential for surgeons to understand not only the normal anatomy of the biliary tract but anatomical variations to avoid iatrogenic injuries [1]. In this article, the authors describe an unusual anatomic variation of the gallbladder that may manifest as biliary colic, combined with two well described vascular anomalies.

The function of the gallbladder is to store and concentrate bile, required for the effective absorption of fat-soluble substances in the small intestine. Bile is produced in the liver, travels through the right and left hepatic ducts to the common hepatic duct and from here bile passes through the cystic duct to be stored in the gallbladder. The volume of a normal gallbladder is reported to be 30–60 ml [2,3]. The lumen of the gallbladder itself usually consists of a single valveless chamber. When chyme enters the small intestine, cholecystokinin is released and stimulates both the contraction of the gallbladder and the relaxation of the sphincter of Oddi, the muscular sphincter at the junction of the hepatopancreatic ampulla with the duodenum. In a normal gallbladder, contraction of its walls causes the excretion of bile back through the cystic duct and the common bile duct on its way to the duodenum [2]. When this process is interrupted, most often due to a stone or tumor in the gallbladder or along its path of drainage, the gallbladder can become inflamed, and may present with symptoms including right upper quadrant abdominal pain and jaundice. Such an obstruction has been hypothesized to act as a valve-like mechanism upon contraction, leading to a pressure-induced expansion of the gallbladder and subsequently biliary colic.

Published descriptions of anatomical variations of the biliary system include abnormal attachments, partial and complete duplications, diverticula, and septa [3]. A transverse septum has rarely been described. Kuznetsov et al. published a case report documenting the presentation and subsequent removal of an extremely large gallbladder that was divided by a complete septation into two separate chambers: a smaller proximal chamber that contained bile and two cholesterol stones, and a larger mucus filled chamber [4]. The development of a “giant gallbladder” is hypothesized to result from enlargement caused by a valve-like mechanism due to a stone, tumor, or a “wandering gallbladder” (i.e. a gallbladder that is freely floating, suspended only by its attachment to the cystic duct and its mesentery) [4]. Deutsch et al. described four cases of congenital septa of the gallbladder that manifested in symptoms resembling cholelithiasis [5]. In addition to these congenital abnormalities, adenomyomatosis of the gallbladder often presents with the appearance of transverse septa, as the overgrowth of mucosa lining the gallbladder wall causes an “hourglass” configuration [6,7].

Other well-described anomalies of the biliary system and hepatic vasculature include duplications of the gallbladder, anomalous left hepatic arteries, accessory left hepatic arteries, and anomalous origins of the right hepatic artery [3]. Vascular anomalies are moderately common variations with an aberrant right hepatic artery occurring in 3.7% of individuals and an accessory left hepatic artery occurring in 3.2% of individuals [8]. This article describes the complex case of a partial transverse septum in a markedly enlarged gallbladder with a dilated duct system and several vascular anomalies. The work reported within this manuscript is in line with the SCARE criteria [9].

## 2 Case presentation

**Fig. 1:**
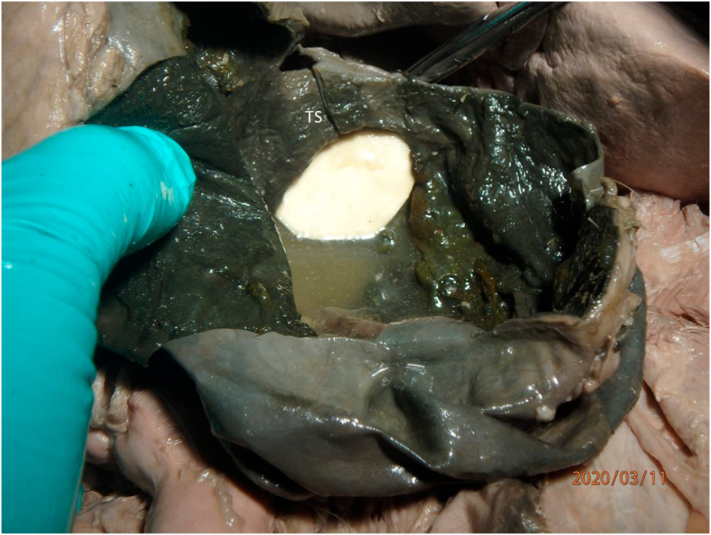
Upon opening the gallbladder, the transverse septum (TS) was visualized. White paper was placed in the smaller cavity of the gallbladder to highlight the opening between the larger, fundal compartment in the foreground and the smaller compartment behind the paper.

**Fig. 2:**
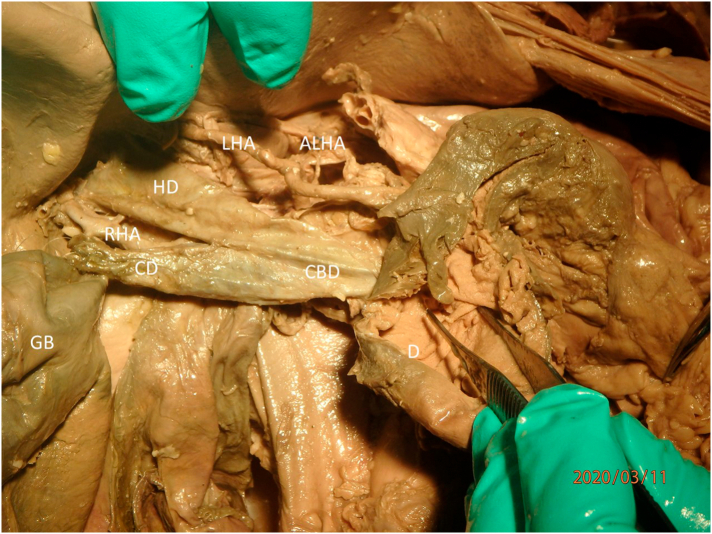
Dissection of the hepatoduodenal ligament revealed an enlarged ductal system, an aberrant right hepatic artery (RHA), and an accessory left hepatic artery (ALHA). GB: gallbladder; CD: cystic duct; HD: hepatic duct; CBD: common bile duct; D: duodenum; LHA: left hepatic artery.

During the dissection of a 91-year-old female cadaver, whose cause of death was reported as Alzheimer's disease, an enlarged gallbladder filled with approximately 350 ml of thin bilious fluid was identified. This gallbladder was normally situated in the fossa for the gallbladder. Upon opening, a prominent transverse partial septation in the body was found. This partial septum was located near the infundibulum (Hartmann's pouch) and this divided the gallbladder into a smaller chamber near the opening of the cystic duct and a larger chamber near the fundus (Fig. 1). There was a one centimeter opening between the two chambers allowing communication of fluid. No stones, mucus, or obstructive masses were found in the gallbladder or along its path of drainage into the duodenum. The diameter of the biliary duct system appeared enlarged; the cystic duct measured 12 mm (normal 2-3 mm), the hepatic duct 18 mm (normal 6 mm) and the common bile duct 21 mm (normal 7 mm), in the collapsed state [3,10]. The common bile duct, near its termination, measured 7 mm with a patent opening into the pancreaticobiliary antrum (Fig. 2). No anatomical explanation for the enlarged ductal system was found. It is possible that the patient passed a gallstone prior to death, causing a constriction at the pancreaticobiliary antrum and subsequent dilation of the biliary tree, but this is only conjecture. The gallbladder wall was of normal thickness and had no evidence of past inflammation in the form of adhesions or other structural changes. The liver appeared grossly normal. The authors hypothesize this septal abnormality was congenital and during life may have caused symptoms of biliary colic due to the septum intermittently obstructing the cystic duct during contraction of the gallbladder; this may have resulted in incomplete emptying of the gallbladder and subsequent enlargement.

**Fig. 3:**
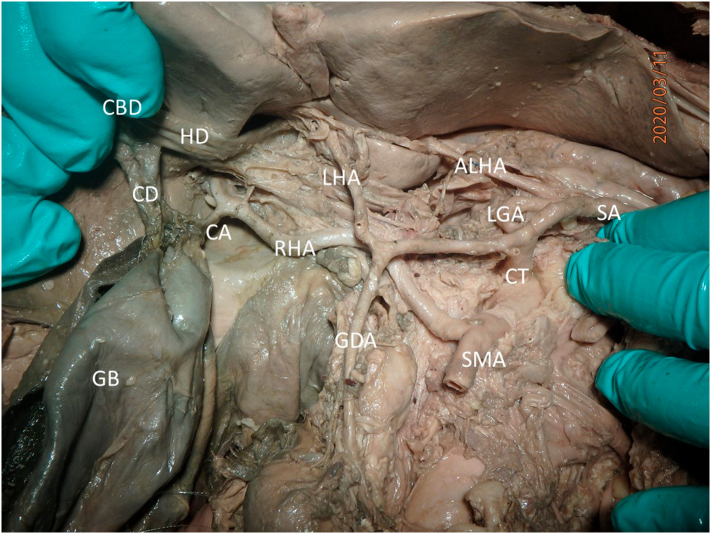
Reflection of the common bile duct (CBD), hepatic duct (HD), and the cystic duct (CD) revealed the right hepatic artery (RHA) as a branch off the superior mesenteric artery (SMA). Additionally, an accessory left hepatic artery (ALHA) was seen branching from the left gastric artery (LGA). CT: celiac trunk; CHA: common hepatic artery; SA: splenic artery; LHA: left hepatic artery; GDA: gastroduodenal artery; GB: gallbladder; CA: cystic artery.

**Fig. 4:**
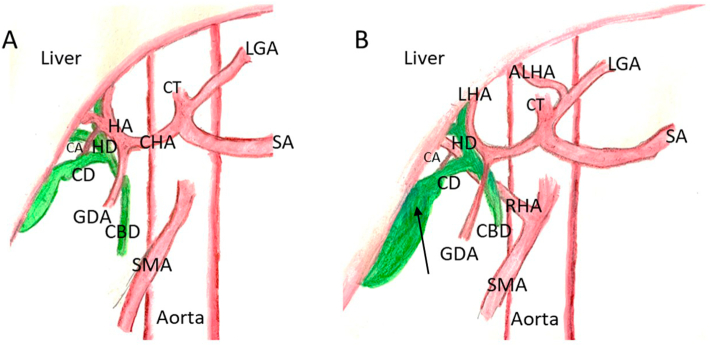
A: Normal anatomy of biliary system and arterial supply. B: Variations observed in this case study. The black arrow demonstrates the approximate location of the partial transverse septum. CA: cystic artery; HA: hepatic artery; CHA: common hepatic artery; HD: hepatic duct; CD: cystic duct; CBD: common bile duct; SMA: superior mesenteric artery; GDA: gastroduodenal artery; RHA: right hepatic artery; LHA: left hepatic artery; CT: celiac trunk; ALHA: accessory left hepatic artery; LGA: left gastric artery; SA: splenic artery.

In addition to the enlarged gallbladder, an aberrant right hepatic artery and an accessory left hepatic artery were found. The left hepatic artery was normally located but there was also an accessory left hepatic artery originating from the left gastric artery. The right hepatic artery originated from the superior mesenteric artery (Fig. 3, Fig. 4). To our knowledge, this combination of a transverse septum with such vascular anomalies has not been reported in the literature (Fig. 4). The vascular variations are likely unrelated to the enlargement of the gallbladder, as there has been no record of these variations causing enlargement of the gallbladder nor development of a partial transverse septum.

## 3 Discussion and conclusions

With the significant annual involvement of biliary disease in the United States, it is clinically relevant to understand and appreciate anatomical variations of the hepatobiliary ductal and vascular system to avoid iatrogenic injuries. While most biliary symptoms are due to gallstones, other causes must be considered and treated surgically when they manifest with symptoms. Our cadaver demonstrated an enlargement of the gallbladder, nearly ten times the normal size, with additional enlargement of the extrahepatic ductal system as well as vascular anomalies. Due to the absence of any signs of previous inflammation or stone formation and a normal attachment to the liver, we conjecture the anomalies seen in this case were congenital and likely resulted in mild or no symptoms of biliary colic. We hypothesize that the expansion of the gallbladder in this case was likely caused by the unique nature of its partial septation. It is possible that upon contraction, a seal may have formed between the septation and the wall of the gallbladder, closing off the larger chamber but still allowing for the smaller chamber to excrete bile into the cystic duct. Communication between the chambers would still be possible between contractions, which explains the homogenous nature of the fluid. It is possible that a transverse septum in the gallbladder could remain asymptomatic; however, if the septum were to intermittently obstruct the opening into the cystic duct, symptoms consistent with cholelithiasis could manifest and cholecystectomy may be considered based on the severity of the patient's symptoms. As this case demonstrates, knowledge of anatomical variations of the gallbladder is clinically essential in correctly diagnosing and treating biliary diseases. While many anatomical variations have been reported in this region, this is the first case to describe a transverse septum in the gallbladder in conjunction with an enlarged ductal system and numerous vascular anomalies. There is not enough evidence to determine any association between the vascular anomalies and gallbladder septum seen in this case. Therefore, the authors emphasize precautions should be taken during cholecystectomy in light of these novel anatomical variations.

## Sources of funding

This research did not receive any specific grant from funding agencies in the public, commercial, or not-for-profit sectors.

## Ethics approval and consent to participate

UTHSCSA Office of IRB deemed this cadaveric case study to be non-regulated research (#HSC20210224N).

## Consent for publication

Consent for publication was waived. The body donor used in this study was donated to the Body Donation Program at UT Health San Antonio for education and research. UT Health San Antonio is a member institution of the State Anatomical Board of Texas. All donations to the program are regulated and cared for under the statutes and guidelines of the State of Texas.

## Authors' contributions

PB: Data Curation, Investigation, Methodology, Writing; KCB: Data Curation, Investigation, Methodology, Writing; MB: Data Curation, Investigation, Methodology, Writing; HLN: Project Administration, Writing; APO: Data Curation, Investigation, Methodology, Conceptualization, Project Administration, Supervision, Writing.

## Authors' information

PB and KCB are current first year medical students at UTHSCSA. MB and HLN are Assistant Professor's in the Department of Cell Systems in Anatomy at UTHSCSA. APO (corresponding author, guarantor) is an Associate Professor in the Department of Cell Systems in Anatomy at UTHSCSA.

## Availability of data and materials

No datasets were generated or analyzed during the current study, therefore data sharing is not applicable. Provenance and peer review. Not commissioned, externally peer-reviewed.

## Declaration of competing interest

The authors declare that they have no competing interests.
